# Protocol for a single-blind randomized clinical trial to test the efficacy of bilateral transcranial magnetic stimulation on upper extremity motor function in patients recovering from stroke

**DOI:** 10.1186/s13063-023-07584-7

**Published:** 2023-09-22

**Authors:** Yuan Huang, Ruizhu Lin, Hongyu Li, Yujuan Xu, Fubao Tian, Liangchen Ma, Xiaoli Liu, Shuming Ma, Xiaolong Li, Zheying Lai, Chuanping Bai, Weichun He, Qi Ma, Jingkai Wang, Ning Zhu

**Affiliations:** Department of Rehabilitation Medicine, General Hospital of Ningxia Medical University, Yinchuan, Ningxia China

**Keywords:** Stroke, rTMS, Combined, Plasticity, Cortical excitability, Serum factor

## Abstract

**Background:**

No consensus currently exists regarding the optimal protocol for repetitive transcranial magnetic stimulation (rTMS) treatment of upper-extremity motor dysfunction after stroke. Studies have shown that combined low- and high-frequency stimulation (LF-HF-rTMS) of the bilateral cerebral hemispheres is more effective than sham stimulation or stimulation of one cerebral hemisphere alone in treating motor dysfunction in the subacute stage of stroke. The efficacy of this protocol in the convalescence phase of stroke has rarely been reported, and its mechanism of action has not been clarified. In this study, we designed a prospective, single-blind, randomized controlled trial to investigate the efficacy and safety of different stimulation regimens for the treatment of upper extremity motor disorders in patients with convalescent stage stroke and aimed to explore the underlying mechanisms based on biomarkers such as brain-derived neurotrophic factor (BDNF).

**Methods:**

Seventy-six subjects will be randomly divided into combined, low-frequency, high-frequency, and control groups based on the proportion of 1:1:1:1, with 19 cases in each group. All groups will have conventional rehabilitation, on top of which the combined group will receive 1 Hz rTMS in the unaffected hemisphere and 10 Hz rTMS in the affected hemisphere. The low-frequency group will be administered 1 Hz rTMS in the unaffected hemisphere and sham stimulation in the contralateral hemisphere. The high-frequency group will be administered 10 Hz rTMS in the affected hemisphere and contralateral sham stimulation. The control group will receive bilateral sham stimulation. Assessments will be performed at baseline, after 2 weeks of treatment, and at post-treatment follow-up at week 6. The primary outcomes are FMA-UE (Fugl-Meyer assessment-upper extremity), latency, and serum BDNF levels. The secondary outcomes are the National Institute of Health Stroke Scale (NIHSS), Brunnstrom staging (BS), modified Ashworth scale (MAS), Modified Barthel Index (MBI), central motor conduction time (CMCT), precursor proteins of mature BDNF (proBDNF), and matrix metalloproteinase-9 (MMP-9) levels. Adverse events, such as headaches and seizures, will be recorded throughout the study.

**Discussion:**

The findings of this study will help develop optimal stimulation protocols for motor recovery in stroke patients and identify biomarkers that respond to post-stroke motor rehabilitation, for better guidance of clinical treatment.

**Trial registration:**

The study protocol was passed by the Medical Research Ethics Committee of the General Hospital of Ningxia Medical University on January 1, 2022 (no. KYLL-2021–1082). It was registered into the Chinese Clinical Trials Registry on May 22, 2022 (no. ChiCTR2200060201). This study is currently in progress.

**Graphical Abstract:**

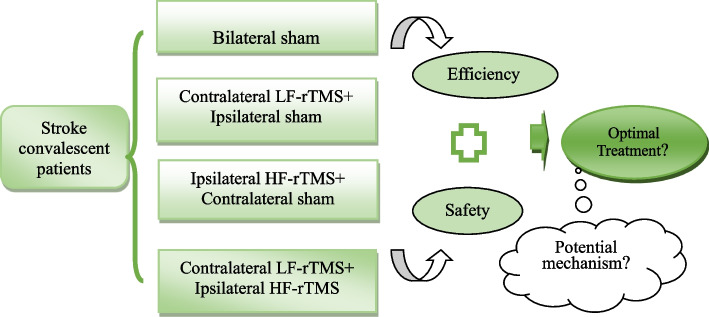

**Supplementary Information:**

The online version contains supplementary material available at 10.1186/s13063-023-07584-7.

## Background

Stroke is an acute condition resulting from the disruption of blood circulation to the brain, falling under the category of non-communicable disease. According to the Global Burden of Disease (GBD) 2019 report by the World Health Organization [1], stroke is the second leading cause of death worldwide and is associated with a significant rate of disability. If the current trend persists, the projected figures indicate that by 2050, there will be approximately 200 million stroke survivors and 13 million annual stroke-related deaths on a global scale [1]. Up to 85% of stroke patients suffer from upper limb dysfunction [2], which severely impacts their daily life and work, imposing substantial limitations and resulting in a considerable financial burden on society [3].

Current conventional upper-limb rehabilitation methods include central interventions with mirror therapy, motor imagery therapy, peripheral interventions with physical factors, and compulsory movement therapy. However, these conventional methods have some limitations due to the high demands on the patient’s consciousness and subjective motivation with long treatment periods. Thus, it is necessary to develop a fast, efficient, and convenient method to improve upper-limb motor function.

In recent years, repetitive transcranial magnetic stimulation (rTMS) has prevailed in neurorehabilitation as a feasible, safe, and painless neurophysiological technique [4]. Studies have shown that neuroplasticity-induced cortical reorganization is an essential regulatory process in motor function recovery after stroke [5], and substantial research has shown that rTMS can promote brain plasticity [6].

The current rTMS treatment protocols for stroke have diverse forms [7], ranging from initial unilateral hemisphere stimulation with low- or high-frequency rTMS alone, TMS combined with other rehabilitation therapies, to low-frequency rTMS stimulation of the unaffected hemisphere together with high-frequency rTMS of the affected hemisphere. The first two protocols have been studied extensively, while less research has been conducted on the latter.

The clinical trial by Long et al. demonstrated that combined 1 Hz and 10 Hz rTMS was effective for the recovery of upper limb motor function in patients with acute stroke [8]. Chen et al. also found that a combination of low- and high-frequency rTMS could synergistically enhance motor function and cortical excitability in patients with subacute stroke [9]. Nevertheless, few studies have compared the effects of this protocol on upper-limb motor deficits in patients with stroke in the convalescent stage.

In addition, the mechanism of action of rTMS in post-stroke motor function remains unclear. It is commonly believed that low-frequency (1 Hz) rTMS inhibits neuronal excitation in the unaffected hemisphere, whereas high-frequency (> 1 Hz) rTMS induces facilitation in the affected hemisphere [10], thereby impacting intracerebral metabolism and neuroelectrical activity. Biomarkers are usually used as measurable objective indicators to analyze therapeutic efficacy [11].

Brain-derived neurotrophic factor (BDNF), a member of the neurotrophin family, is associated with the regulation of neuronal reorganization and is involved in the pathogenesis of many neurological diseases [12]. BDNF exists in two forms, mature BDNF and BDNF precursor protein (proBDNF), which are functionally opposite [13]; proBDNF can be converted to BDNF by matrix metalloproteinase-9 (MMP-9) [14]. In an animal study, BDNF was revealed to be able to cross the blood–brain barrier (BBB) [15], and its levels in serum correlated well with those in brain tissue [16]. Notably, the concentration of BDNF and pro-BNDF in peripheral blood is considered to be representative of its function in the brain, and serum levels may to some extent reflect brain function [17–19]. Therefore, assessing their levels in individual serum sample will have clinical and scientific implications. In recent years, there are many studies on the relationship between BDNF and stroke, but little attention has been paid to proBDNF and MMP-9.

This trial aims to investigate the clinical efficacy of LF-HF-rTMS on upper extremity motor function in convalescent stroke patients and to explore the mechanisms underlying its therapeutic effects. This study will assess the following: significant changes in clinical behavioral assessments and cortical excitatory parameters in patients with convalescent stroke after 10 interventions with rTMS, compared with that by sham and unilateral hemispheric stimuli. In addition, we will measure the concentrations of neuroplasticity mediators, serum BDNF, pro-BDNF, and MMP-9, and analyze their correlation with behavioral improvement.

## Methods

### Study design

**Fig. 1:**
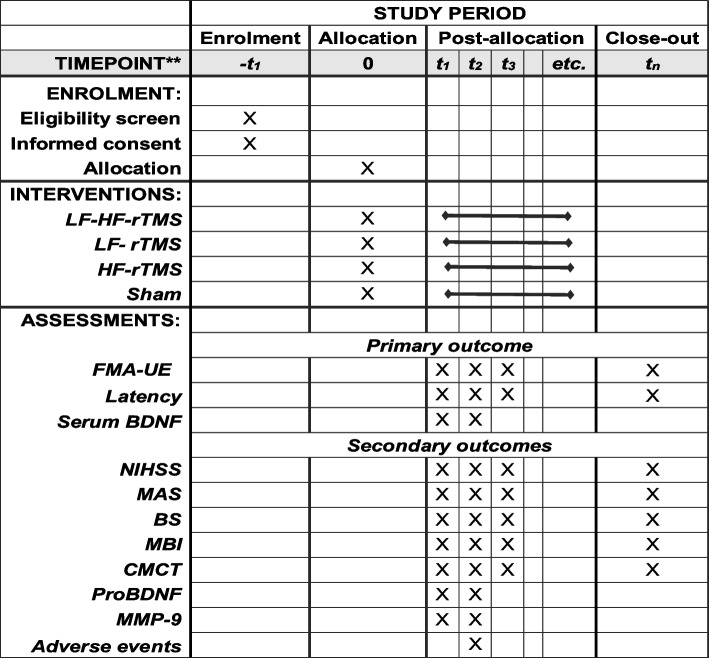
The SPIRIT schedule. LF-HF-rTMS, low- and high-frequency rTMS; LF-rTMS, low-frequency rTMS; HF-rTMS, high-frequency rTMS; FMA-UE, Fugl-Meyer assessment-upper extremity; BDNF, brain-derived neurotrophic factors; NIHSS, National Institutes of Health Stroke Scale; MBI, modified Barthel index; MAS, modified Ashworth scale; BS, Brunnstrom staging; CMCT, central motor conduction time; ProBDNF, precursor proteins of mature BDNF; MMP-9, matrix metalloproteinase-9

This is a prospective, single-blind, randomized controlled clinical trial with hidden assignment and intent-to-treat analysis. We will recruit 76 patients with upper extremity motor deficits after stroke in convalescence and then randomly and equally assign them to LF-HF-rTMS, low-frequency, high-frequency, and sham stimulation groups. Subjects will be assessed for clinical behavior, neurophysiology, and serologic testing at baseline and after 2 weeks; further follow-up assessment of clinical behavior will be performed after 6 weeks (Fig. 1).

The protocol was written based on the Standard Protocol Item: Recommendation for Interventional Trials (SPIRIT, S1 File) checklist [20]. This study is in accordance with the latest revised version of the Declaration of Helsinki 2013.

### Recruitment

**Fig. 2:**
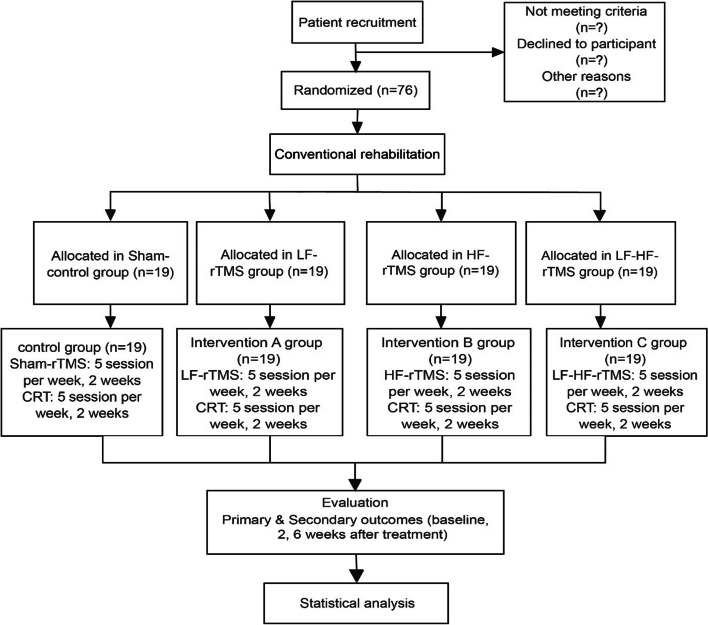
Recruitment of participants and flow chart. CRT: Conventional rehabilitation treatment

We will recruit 76 stroke patients with upper-limb movement disorders during recovery from the inpatient unit of the Department of Rehabilitation Medicine at the General Hospital of Ningxia Medical University (Fig. 2). Stroke patients account for the majority of our unit’s patients, and the inpatient period is 2 weeks, which is sufficient to ensure the required number of recruits and interventions. Patients will be recruited through various forms, including WeChat promotion, hospital posters, verbal presentations by physicians, and community outreach. Participating patients enjoy a privileged policy in which all assessments and neurophysiological examinations are free of charge during the treatment period, and registration fees and transportation costs are reimbursed during follow-up visits. Patients who agree to join this study will be recruited, and demographic information and assessment data will be collected and monitored by the attending physician.

### Inclusion criteria

They will be recruited for the following criteria: (1) those with diagnosis in accordance with the relevant diagnostic criteria for cerebral infarction and cerebral hemorrhage in the “Chinese Guidelines for the Prevention and Treatment of Cerebrovascular Diseases” [21], confirmed by cranial CT or MRI to affect unilateral cerebral hemisphere; (2) disease duration of 30–180 days, and patients with stable vital signs, clear consciousness, and willingness to cooperate; (3) those with the first incidence of stroke; (4) those aged 30–75 years; (5) upper-limb Brunnstrom stages 2–5; and (6) patients and family members are willing to sign the informed consent form.

### Exclusion criteria

These patients will be excluded in case of the following conditions: (1) those with unstable disease, progressive stroke, malignant progressive hypertension, or cerebral hemorrhage secondary to cerebral infarction; (2) those with apparent indications of increased intracranial pressure; (3) those with contraindications to transcranial magnetic stimulation therapy, such as epilepsy, implanted pacemakers or drug pumps, metal objects in the skull or eyes; (4) those with combined cognitive dysfunction; (5) those with combined malignancy; and (6) those with severe lung, heart, liver, and kidney dysfunctions.

### Patient withdrawal criteria

The patient withdrawal criteria are as follows: (1) patients whose decision changes during the disease, rendering them unsuitable for continued treatment, and (2) other personal reasons.

### Randomization

After meeting the inclusion criteria, 76 subjects will be randomly assigned in a 1:1:1:1 ratio to the LF-HF-rTMS, low-frequency, high-frequency, and sham stimulation groups. The grouping will be performed according to a PC-generated random number, which will be concealed in a sealed envelope. The random sequence will be managed and supervised by specific personnel who have no contact with the subjects and are not included in data collection or analysis.

### Blinding

In this study, numbers 1, 2, 3, and 4 will replace the four groups in the case report form (CRF) table. Subjects will unaware of whether they are in the treatment or sham stimulation groups. In addition, baseline and treatment phase assessments will be implemented by a researcher without knowledge of allocation. Unblinding will be performed at the end of the data analysis for this study.

### Patient public involvement

The public or patients will not be involved in the design of this protocol.

### Intervention

The combined, low-frequency, high-frequency, and control groups will receive microcirculation improvement, hypotensive medication, and conventional rehabilitation treatment, according to their disease characteristics. Based on the treatments, different rTMS stimulation protocols will be administered to the three treatment groups. This study will be conducted by two experienced and trained occupational therapists qualified for rTMS intervention. The other two evaluators will be unaware of the patient grouping. We will remind the participants of the consultation in advance to understand the change of the patient’s condition, treatment status, compliance, and complete the relevant follow-up records.

#### Conventional treatment

1. Pharmacological treatment: All groups of patients will receive the corresponding pharmacological treatment according to the characteristics of their disease, such as blood pressure control, blood glucose, blood lipids, nerve nutrition, and other pharmacological therapies.
2. Conventional rehabilitation treatment: This refers to the guidelines for exercise rehabilitation given in the 2011 edition of the Chinese Stroke Rehabilitation Guidelines [22], which mainly include (a) good limb position and active and passive joint mobility training; (b) core muscle strength training, balance training, and walking training; (c) prevention and treatment of spasticity; and (d) training of manual refinement and daily living self-care ability. Occupational therapy and physical therapy will be administered for 40 min each, twice daily for 2 weeks over a total of 10 days.

#### rTMS treatment

A CCY-I magnetic field stimulator (Irid Medical Equipment New Technology Co., Ltd., Wuhan, China) with a circular coil of 125 mm diameter will be used once daily for 20 min.

**Fig. 3:**
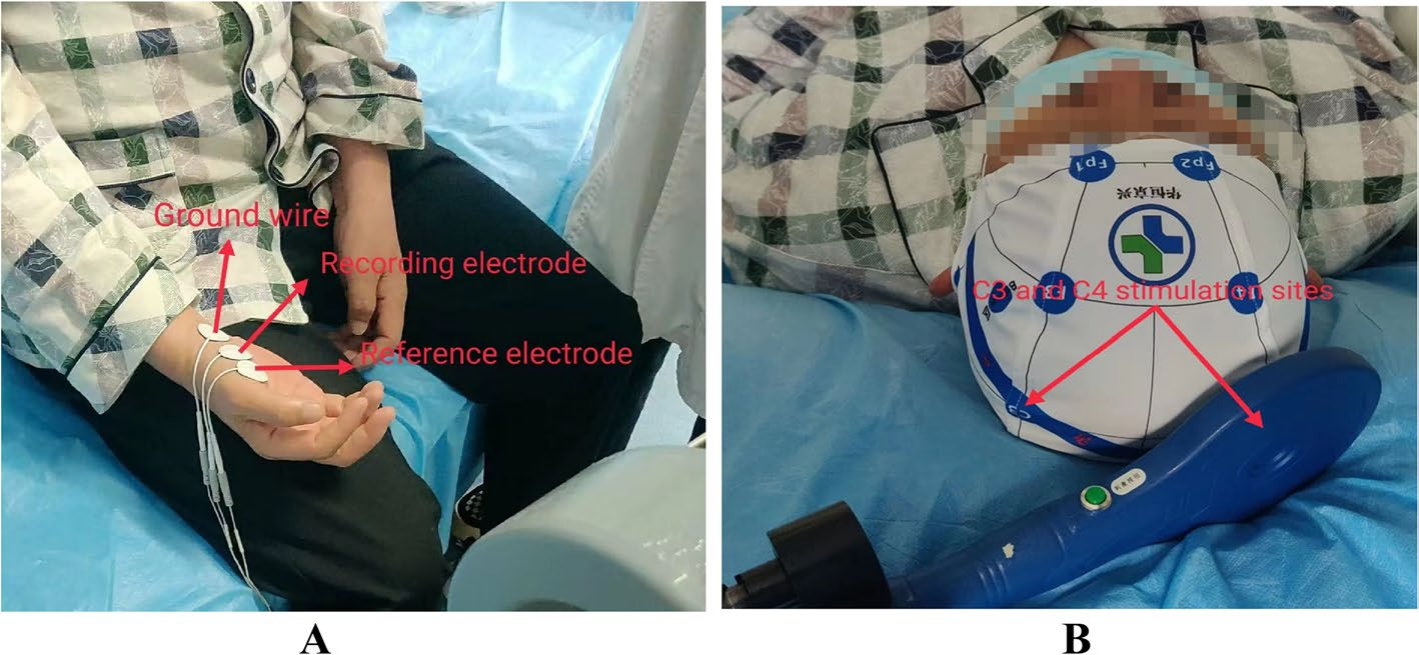
Sketch of the rTMS stimulation protocol. **A** Schematic diagram for attaching electrodes. **B** Diagram of the coil stimulation site

The patient will be placed in a supine position, fully relaxed, with the head held still in a positioning cap. First, the motor evoked potential (MEP) monitoring module will be activated and then connected to the lead. The center of the circular coil will be aligned with a representative area of hand function in the cerebral cortex, such that the coil will be tangential to the skull surface at 45°. Subsequently, a single-pulse magnetic stimulation pattern will be used to observe the responsive movements of the affected fingers. If stimulation of the primary motor cortex (area M1) detects a minimum MEP with an amplitude ≥50 μV in the short thumb adductor muscle at least 5 times out of 10, this point will be the target of the stimulus and the stimulus strength will be the hemispheric resting motion threshold (RMT) (Fig. 3).

#### Stimulation parameters

- Low-frequency combined with high-frequency rTMS group (LF-HF-rTMS group): patients will first undergo low-frequency rTMS treatment on the unaffected side, M1 area, at 1 Hz, 90% RMT, continuous stimulation, 600 pulses for 10 min, then high-frequency rTMS treatment on the affected side, M1 area, at 10 Hz, 90% RMT, intermittent stimulation, 600 pulses for 10 min
- Low-frequency rTMS group: patients will receive stimulation to the M1 region of the contralateral hemisphere, at 1 Hz, 90% RMT, continuous stimulation, 1200 pulses. The affected side will then undergo a 10-Hz sham stimulus, for 20 min
- High-frequency rTMS group: patients will receive stimulation to the M1 area of the affected hemisphere, at 10 Hz, 90% RMT, intermittent stimulation, 1200 pulses. The unaffected side will then undergo a 1-Hz sham stimulus, for 20 min
- Sham stimulation group: patients receive stimulation in both hemispheres under the same set of parameters as the combined group, with the coil at 90° and perpendicular to the scalp without power output, but sounds will be produced

All patients will be strictly treated according to the 2021 TMS operation guidelines [23], and the treatment course will be 2 weeks. Patients’ vital signs and adverse reactions will be closely monitored during treatment, and they will be discharged without discomfort at the termination of the treatment.

### Primary outcomes

1. The Fugl-Meyer upper extremity score scale (FMA-UE) will be employed to evaluate the patient’s upper extremity motion capabilities. It contains elements such as reflex, coordination, stability assessment, and motor assessment of the shoulder to hand and is divided into nine items with a score of 0 points indicating the inability to complete a movement, one point indicating partial completion, and two points indicating successful completion, for a total score of 66. The one who has a smaller score is the one who has severe motor disability.
2. Latency: MEP latency is the time between the onset of stimulus in the motor cortex and the appearance of action potentials in the contralateral target muscle [24]. A stimulus with an intensity of 90% RMT will be applied to the M1 area of the affected hemisphere, and the action-evoked potentials of the contralateral thumb abductor muscle will be recorded, taking three waveforms with good repeatability to record their latency values.
3. Measuring serum biomarkers. Peripheral blood BDNF expression levels will be quantified using human BDNF-ELISA kits (manufactured by ABclonal, RK00074) [25]. Five milliliters of venous blood will be drawn, and the supernatant will then be centrifuged for 10 min at 3500 rpm at 4 °C (Centrifuge 5810 R, Eppendorf, Germany). The samples will be promptly anonymized and stored in a refrigerator at − 20 °C. Approximately 1 week after collection, the frozen samples will be transported to the laboratory to Ningxia Medical Uni’s laboratory and maintained at – 80 °C until analysis.

### Secondary outcomes

1. The National Institute of Health Stroke Scale (NIHSS) is used to evaluate the neurological function state of patients with stroke. This scale includes 11 items. The total score ranges from 0 to 42 points; and the higher the score, the more severe the nerve injury [26].
2. The Brunnstrom staging (BS) scale for assessing movement function improvement in stroke patients. It is divided into stages I–VI; the greater the staging, the superior the restoration of function.
3. Modified Ashworth scale (MAS) to evaluate variations in the patient’s muscle tone on a scale of 0, 1, 1 + , 2, 3, and 4 [27]; the higher the grade, the greater the muscle tone.
4. The Modified Barthel Index (MBI) will be administered to the assessment of activities of daily living [28]. The total score is 100; a score ≤ 50 is classified as severe functional impairment, 50–75 as moderate functional impairment, 75–100 as mild functional impairment, and > 100 as no functional impairment.
5. Central motor conduction time (CMCT) refers to the conduction time from the cerebral cortex to the alpha motor neurons in the anterior horn of the spinal cord. An intensity stimulus of 90% RMT will be applied to the patient’s seventh cervical spine (C7), and transmission time from C7 to the thumb short abductor muscle will be measured to calculate the difference between the transmission time from the scalp to the electrode piece and the transmission time from C7 to the electrode piece [29].
6. Measuring serum biomarkers: the human MMP-9-ELISA kit (produced by ABclonal, China) will be used to quantify the level of MMP-9 expression in peripheral blood, and a human ProBDNF-ELISA kit (Biosensis, USA) will be used to detect the peripheral blood ProBDNF factor concentration [25]. The same protocol will be used to detect BDNF.

### Adverse events and safety monitoring

Adverse events will be monitored by a physical therapist and will include headaches in the stimulation area and hearing loss (due to coil noise). Earplugs will be placed on the patient during stimulation to prevent hearing damage. If localized pain or headache occurs, it is usually mild and can disappear quickly. The most serious side effect that can be triggered by TMS is seizures; however, the probability of occurrence is extremely low. Unsafe effects for TMS use by operators have not been reported since a long time. The patient’s vital signs, including heart rate, blood oxygen, and respiration, will be closely monitored during treatment.

### Sample size

We have calculated the sample size using the PASS 15 software. A literature search was conducted using FMA-UE as the primary indicator [8], specified as a two-sided test with *α* = 0.05 with a degree of certainty (test efficacy1-β) of = 0.8, and a total sample size of *N* = 60 cases was calculated. Considering a 20% dropout rate and the average distribution of each group, the total sample size was 76, with 19 individuals included in each group.

### Data analysis

Data analysis will be performed by the SPSS 26.0 statistical software, and statistical analysis of efficacy will be based on intention-to-treat analysis (ITT) principles.

1. We will check whether the data is normally distributed using the Shapiro Wilk normality test. Nonparametric analysis such as chi-square test will be applied for the count data, and it will be expressed as rate or composition ratio. Nonnormal data will be tested using the Kruskal–Wallis test.
2. Paired *t*-tests will be used for pre- and post-treatment comparisons of groups, one-way ANOVA for inter-group comparisons, and Bonferroni correction for post hoc comparisons between groups.
3. Two-factor repeated measures ANOVA will be employed to investigate the impact of within-group factors (time points) and between-patient factors (subgroups) on outcomes and to analyze main and interaction effects.
4. Pearson correlation analysis will be performed to investigate the correlation between cortical excitability (latency and CMCT) and motor function (FMA), the relationship between serum factors (BDNF, proBDNF, and MMP-9) and motor function, and the correlation between cortical excitability and serum factors.

A *P* value of < 0.05 will be regarded as statistically significant.

### Participant management and data management

Eligible subjects will be screened by dedicated recruitment staff based on the strict criteria. The number of subjects will be distributed sequentially according to the order of inclusion; ineligible subjects will be excluded, the reasons for exclusion will be recorded, and the number counted. The evaluation staff and corresponding study staff must promptly complete the CRF. After reviewing and completing the study report form, the data analyst will make a double entry and the project management staff will manage the original report form in a unified manner. Routine supervision of the data and safety of this study by a monitoring committee which consists of three members from the Clinical Trial Center of the General Hospital of Ningxia Medical University. The test plan and data records will be strictly followed to ensure the authenticity of the operation. The project management team, consisting of two senior and senior physicians, meets twice a month to review the implementation of the trial. The trial steering group and the independent data monitoring and ethics committee met to review the entire operation during the trial. The data management team, monitoring committee, and experimental researchers are independent of each other, and there are no conflicts of interest.

### Confidentiality

The participant’s data will be kept confidential. All clinical sample data (rehabilitation assessment results and serum test results) will be identified and manipulated according to their number. Identifiable information will be encrypted and not disclosed to anyone other than the investigator. The participant’s data will be kept in a locked drawer for investigator reference. After testing, all leftover blood samples will be destroyed.

## Discussion

### Mechanism and importance of rTMS

Stroke is the leading cause of death in China, surpassing heart disease and malignant tumors [28]. Stroke directly affects the motor nerve center and the network structures of both hemispheres away from the diseased cortical area, weakening the control of the cerebral cortex over the corresponding motoneurons that innervate the movements of the upper limbs, thus leading to upper limb paralysis [29]. The interhemispheric inhibition model (IHI) indicates that there is a dynamic equilibrium of mutual inhibition between the healthy hemispheres. However, after stroke, equilibrium is disrupted; the affected hemisphere is hypoexcitable and the contralateral hemisphere is hyperexcitable, resulting in the affected side being inhibited by the contralateral side despite its own impairment. However, LF-rTMS inhibits the excitability of the unaffected motor cortex, while HF-rTMS promotes the excitation of the affected cortex, which can be used to restore the equilibrium between the two sides of the cortex disrupted by brain injury [30].

rTMS is an emerging and non-invasive brain stimulating technique developed in the 1980s to produce depolarization and action potentials in the cerebral cortex [23]. rTMS has been shown to affect the strength of synaptic connections and the efficiency of information transmission as well as to modulate corticospinal excitability and promote neural plasticity by producing long-term potentiation (LTP) and long-term depression (LTD) [5, 6]. Several studies have indicated that not only can TMS be used to predicts the prognosis of stroke patients by determining the integrity of the corticospinal tract using MEP measurements, but rTMS is also effective in improving the levels of motility in the hemiplegic upper limb [31, 32].

### The choice of rTMS stimulus protocol

A recent animal study [33] suggested that high-frequency (20 Hz) rTMS promoted better muscle strength and motor coordination than low-frequency (1 Hz) rTMS in a mouse model of acute and subacute ischemic injury. Askin [34] and Kim et al. [35] also found that, in patients recovering from stroke, single-sided low-frequency and high-frequency rTMS were remarkably effective in improving motor function compared with those in conventional treatment. Considerable researches have confirmed the importance of low- and high- frequency rTMS alone in enhancing the upper limb functionality level after stroke, but clinical efficacy analysis favors the combination of low- and high-frequency rTMS over single stimulation [8, 36].

Long et al. randomly divided 62 early stroke patients into a group who received treatment of the unaffected side at 1 Hz combined with the treatment of the affected side at 10 Hz, a low-frequency group, and a sham stimulation group; they found the combined group to be more effective than the low-frequency group in bettering exercise levels in the upper extremities and that patients had better tolerability [8]. New research has also shown that double-target stimulation in patients with generalized anxiety disorder has stronger clinical efficacy than single-target stimulation in improving anxiety, depression, and insomnia [37]. However, there is a lack of research adopting combined applications as a treatment for paraplegic upper limb locomotion, especially during the recovery period. Therefore, the exact clinical efficacy of this combination still needs to be verified with a large number of studies. This trial will compare the clinical efficacy of the LF-HF-rTMS stimulation protocols on upper extremity dyskinesia in patients recovering from stroke.

### The choice of biomarkers

Neuroplasticity-induced cortical reorganization is a crucial regulatory process in the recovery of motor function in hemiplegic patients [38, 39]. As neuromodulators, BDNF, pro-BDNF, and MMP-9 are closely related to the recovery of neurological and psychiatric diseases such as stroke [12]. BDNF binds to the TrkB receptor to trigger neuronal survival and enhance neuronal plasticity and neurogenesis, whereas proBDNF binds to the apoptotic receptor p75 to cause neuronal death [5]. Non-randomized experimental research has shown that low-frequency rTMS modulates changes in blood levels of BDNF, pro-BDNF, and MMP-9 in patients with moderate paralysis, promoting upper limb motor function [40]. Another study showed that high-frequency rTMS combined with cognitive training was effective in increasing serum BDNF concentrations in post-stroke cognitive impairment during stroke recovery [41]. The study protocol will investigate the effects of LF-HF-rTMS on serum factors in patients recovering from stroke.

### Limitations and prospects

One limitation of this study is that it is a small sample trial and the follow-up duration is short. In the future, the sample size will be increased for multi-center trials, and the long-term effects of this scheme will be studied. In addition, because of the large inter-individual variation in cortical mapping, we relied only on surface markers to identify the target cortices, which may lead to localization bias. In the future, functional neuroimaging techniques (such as functional magnetic resonance imaging and functional near infrared spectroscopy) will be combined to guide further research.

## Conclusion

Comprehensive assessment of clinical symptoms and functional scales for stroke patients combined with biological markers can be used to accurately assess the functional prognosis of stroke patients [42]. In this study, FMA-UE, latency, and serum BDNF levels will be used as the main indicators, as well as subjective and objective indicators such as NIHSS, BS, MAS, MBI, CMCT, pro-BDNF, and MMP-9, which will be used to comprehensively evaluate the efficacy of LF-HF-rTMS in enhancing upper limb movement. Based on the changes in nerve electrophysiology and serum factors in patients, we will explore the possible molecular mechanisms of rTMS promoting motor recovery of the upper extremities after stroke. This study is expected to offer new proof for potent protocols and biomarkers for rTMS to augment upper limb motility levels during stroke recovery.

## Trial status

This study started in June 2022, and recruitment and interventions are currently underway. The trial is expected to end in December 2023. Protocol version 1.0 (December 2021).

### Supplementary Information

**Additional file 1.** SPIRIT 2013 checklist.

**Additional file 2. **Ethical Approval Documentation (English).

**Additional file 3. **Ethical Approval Documentation (Chinese).

## Data Availability

All tables, figures, and file information from this study that are not shown in the results section of the article will be made available via the Internet upon request from readers after the publication of this clinical study. These are also available from the first author if the reader so desires.

## Abbreviations

rTMS: Repetitive transcranial magnetic stimulation
LF-HF-rTMS: Combined low- and high-frequency stimulation
FMA-UE: Fugl-Meyer assessment-upper extremity
BDNF: Brain-derived neurotrophic factor
NIHSS: National Institute of Health Stroke Scale
BS: Brunnstrom staging
MAS: Modified Ashworth scale
MBI: Modified Barthel Index
CMCT: Central motor conduction time
proBDNF: Precursor proteins of mature BDNF
MMP-9: Matrix metalloproteinase-9
BBB: Blood-brain barrier
CRF: Case report form
IHI: Interhemispheric inhibition model
LTP: Long-term potentiation
LTD: Long-term depression
