# Supplementary material for: Protocol for a single-blind randomized clinical trial to test the efficacy of bilateral transcranial magnetic stimulation on upper extremity motor function in patients recovering from stroke
Source: Trials. 2023 Sep 22;24:601. doi: 10.1186/s13063-023-07584-7 (PMC10515042; doi:10.1186/s13063-023-07584-7)
Supplement: Supplementary file 2 — Additional file 2. Ethical Approval Documentation (English). [file 13063_2023_7584_MOESM2_ESM.pdf]

Approval of Medical Research Ethics Review Committee of General Hospital of Ningxia Medical Universit

Project name	Explore the mechanism of combined application of rTMS on the efficacy of upper limb motor function and neuroplasticity after stroke based on serum BDNF-like related factors			Ethics No	KYLL-2021-1082		
Project leader	Zhu Ning	professional title	archiater	contact number	13995310532	Department	Rehabilitation Department
Person in charge Research unit:	General Hospital of Ningxia Medical University						
Cooperative research unit:	nothing						
Research time :	2022-01-01		至 2023-12-22				
Whether digital medicine (3D printing) is involved	◉ Yes ○ No						
Whether stem cells and regenerative medicine are involved	◉ Yes ○ No						
Source of research project:							
☐ Vertical research (government support) ☐ Association/Foundation ☐ Company ☑ Independent ☐ others							
Research funding sponsor:	other						
Test object:	☐ Animal ☐ cell ☐ Medical record data/medical image data ☑ Human tissue specimens and samples						
Review comments:							
☑ It meets the ethical requirements and can be tested according to this plan.<br>☐ If it does not meet the ethical requirements, please revise it and then report it to the Ethics Committee for review.							
Informed consent:	◉ Yes ○ No						
Methods for obtaining informed consent:	◉ Appropriate ○ inappropriate						

2023-02-28 朱宁 宁医大总院

Medical Research Ethics Review Committee of General Hospital of Ningxia Medical University	
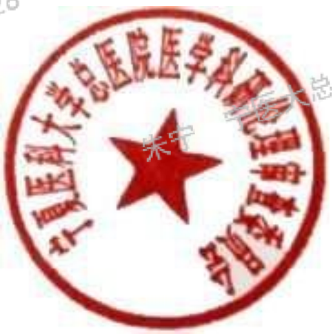	
Attachment upload:	
Informed consent	Informed consent form (1) (1). doc (47KB)
Study protocol	Study protocol.docx (59KB)
Technology roadmap	Technical Roadmap. docx (192KB)
Slide PPT	Medical Research Ethics Approval Application Report of General Hospital of Ningxia Medical University ppt.ppt (169KB)
Approval opinions of the committee	[Agree]<br>[Agree]<br>[Agree] Agree<br>[Agree] Agree<br>[Agree] Agree<br>[Agree]<br>[Agree] Agree<br>[Agree]<br>[Agree]<br>[Agree]<br>[Agree]

2023-02-28 朱宁 宁医大总院
