# Supplementary material for: Protocol for a single-blind randomized clinical trial to test the efficacy of bilateral transcranial magnetic stimulation on upper extremity motor function in patients recovering from stroke
Source: Trials. 2023 Sep 22;24:601. doi: 10.1186/s13063-023-07584-7 (PMC10515042; doi:10.1186/s13063-023-07584-7)
Supplement: Supplementary file 3 — Additional file 3. Ethical Approval Documentation (Chinese). [file 13063_2023_7584_MOESM3_ESM.pdf]

# 宁夏医科大学总医院医学科研伦理审查委员会批准书

| | | | |
|---|---|---|---|
| 项目名称 | 基于血清BDNF类相关因子探讨联合应用rTMS对脑卒中后上肢运动功能疗效及神经可塑性的机制研究 | 伦理编号 | KYLL-2021-1082 |

| | | | | | | | |
|---|---|---|---|---|---|---|---|
| 项目负责人 | 朱宁 | 职称 | 主任医师 | 联系电话 | 13995310532 | 科室 | 康复一病区 |

| | | | |
|---|---|---|---|
| 负责人研究单位: | 宁夏医科大学总医院 | | |
| 合作研究单位: | 无 | | |
| 研究时间: | 2022-01-01 | 至 | 2023-12-22 |
| 是否涉及数字医学(3D打印)相关技术 | ○是 ◉否 | | |
| 是否涉及干细胞与再生医学 | ○是 ◉否 | | |

研究项目来源:

☐纵向研究（政府支持） ☐协会/基金会 ☐公司 ☐横向合作 ☐多中心研究 ☑自主 ☐其他

| | |
|---|---|
| 研究经费资助单位: | 其他 |
| 试验对象: | ☐动物 ☐细胞 ☐病历资料/医学影像资料 ☑人体组织标本和样本 |

评审意见:

☑符合伦理学要求，可以按照此方案进行试验。
☐不符合伦理学要求，请修改后再报伦理委员会审查。

| | |
|---|---|
| 知情同意书: | ◉有 ○无 |
| 获取知情同意书方法: | ◉适当 ○不适当 |

宁夏医科大学总医院医学科研伦理审查委员会

附件上传:

| | |
|---|---|
| 知情同意书 | 知情同意书(1)(1).doc(47KB) |
| 试验方案 | 治疗方案.docx(59KB) |
| 技术路线图 | 技术路线图.docx(192KB) |
| 幻灯PPT | 宁夏医科大学总医院医学科研伦理审批申请汇报ppt.ppt(169KB) |

朱宁 宁医大总院 2023-02-28

| | |
|---|---|
| 委员会审批意见 | 【同意】<br><br>【同意】<br><br>【同意】 同意<br><br>【同意】 同意<br><br>【同意】 同意<br><br>【同意】<br><br>【同意】 同意<br><br>【同意】<br><br>【同意】<br><br>【同意】<br><br>【同意】 |

2023-02-28 朱宁 宁医大总院
2023-02-28 朱宁 宁医大总院
朱宁 宁医大总院
